# Exploring the cellular network of metabolic flexibility in the adipose tissue

**DOI:** 10.1186/s12263-018-0609-3

**Published:** 2018-07-05

**Authors:** Samar H. K. Tareen, Martina Kutmon, Michiel E. Adriaens, Edwin C. M. Mariman, Theo M. de Kok, Ilja C. W. Arts, Chris T. Evelo

**Affiliations:** 1 Maastricht Centre for Systems Biology (MaCSBio), Maastricht University, Maastricht, the Netherlands; 2 Department of Bioinformatics - BiGCaT, NUTRIM School of Nutrition and Translational Research in Metabolism, Maastricht University, Maastricht, the Netherlands; 3 Department of Human Biology, NUTRIM School of Nutrition and Translational Research in Metabolism, Maastricht University, Maastricht, the Netherlands; 4 Department of Toxicogenomics, GROW School of Oncology and Developmental Biology, Maastricht University, Maastricht, the Netherlands; 5 Department of Epidemiology, CARIM School for Cardiovascular Diseases, Maastricht University, Maastricht, the Netherlands

**Keywords:** Obesity, Metabolic flexibility, Regulation, Networks, Pathways, Metabolism

## Abstract

**Background:**

Metabolic flexibility is the ability of cells to change substrates for energy production based on the nutrient availability and energy requirement. It has been shown that metabolic flexibility is impaired in obesity and chronic diseases such as type 2 diabetes mellitus, cardiovascular diseases, and metabolic syndrome, although, whether it is a cause or an effect of these conditions remains to be elucidated.

**Main body:**

In this paper, we have reviewed the literature on metabolic flexibility and curated pathways and processes resulting in a network resource to investigate the interplay between these processes in the subcutaneous adipose tissue. The adipose tissue has been shown to be responsible, not only for energy storage but also for maintaining energy homeostasis through oxidation of glucose and fatty acids. We highlight the role of pyruvate dehydrogenase complex–pyruvate dehydrogenase kinase (*PDC-PDK*) interaction as a regulatory switch which is primarily responsible for changing substrates in energy metabolism from glucose to fatty acids and back. Baseline gene expression of the subcutaneous adipose tissue, along with a publicly available obesity data set, are visualised on the cellular network of metabolic flexibility to highlight the genes that are expressed and which are differentially affected in obesity.

**Conclusion:**

We have constructed an abstracted network covering glucose and fatty acid oxidation, as well as the *PDC-PDK* regulatory switch. In addition, we have shown how the network can be used for data visualisation and as a resource for follow-up studies.

**Electronic supplementary material:**

The online version of this article (10.1186/s12263-018-0609-3) contains supplementary material, which is available to authorized users.

## Background

Metabolic flexibility is defined as the ability of an organism to adapt its substrate for energy production in cellular respiration, based on the availability of the substrates [1]. The primary substrates are glucose and fatty acids, which are converted to acetyl-coenzyme A (acetyl-CoA) for use in the tricarboxylic acid cycle (TCA cycle). Cellular respiration for most tissues and organs utilises only one energy substrate at a given time; glucose during the fed state and fatty acids during the fasted state (exceptions include the brain for example). However, it has been observed that under stress and severe energy deprivation conditions, this exclusivity can be broken and both glucose and fatty acids are consumed for energy production [1].

Given that metabolic flexibility is associated with maintaining a dynamic and shifting balance between the two sources of energy, it may have a prominent role in the development of metabolic diseases and associated conditions. The inability or impairment of the organism to change its source as per requirements is called metabolic inflexibility. A number of recent studies have started focusing on its association with conditions pertaining to malfunctioning metabolism, including obesity, type 2 diabetes mellitus (T2DM), cardiovascular diseases (CVD) and metabolic syndrome (MetS) [2–5]. Considering the implication of metabolic flexibility in disease development, we focus on curating the underlying cellular/molecular mechanisms in this study, specifically in the adipose tissue as several adipose tissue gene expression markers have linked it with reduced metabolic flexibility [6].

Adipose tissue holds a central role in metabolic flexibility and energy metabolism with major regulatory mechanisms and roles, both tissue- and organism-wide [7, 8]. Although adipose tissue stores the majority of the fat in the body, most of the fat is synthesised de novo by the liver. The adipose tissue ends up storing both the synthesised fat released by the liver, as well as dietary fat [9]. In addition, the adipose tissue only takes up 10–15% of circulating glucose [10]. However, this interplay and balance between glucose uptake as well fatty acid uptake and later release is the result of metabolic flexibility in the adipose tissue. Indeed, metabolic inflexibility in the adipose tissue has been known to cause impaired adipokine signalling, as well as impaired non-esterified fatty acid (NEFA) clearance from circulation, triggering NEFA-mediated signalling cascades in other tissues (reviewed in [11, 12]). Thus, the impairment of metabolic flexibility in the adipose tissue can cause systemic effects with regard to energy provision and related processes.

In this review, we summarise the cellular mechanisms pertaining to metabolic flexibility in a network of interacting molecular species and processes. The major benefit of this approach is that it allows further study of the various cellular processes involved in metabolic flexibility to pinpoint crucial elements in the said systems. Similar approaches have previously been employed, for example in [13] where data and existing knowledge were collectively used to identify seemingly unrelated processes involved in adipogenesis in culture. In our review, we employ existing knowledge in terms of known pathways to curate a network representing cellular metabolic flexibility in the adipocytes. Subsequently, baseline expression data of the subcutaneous adipose tissue [14] along with expression data from a publicly available obesity dataset [15] are mapped onto the network as a use case showing the expression of the components of the network under baseline/non-diseased and obese conditions.

## Biochemical pathways of metabolic flexibility

In this review, we have curated an abstracted network representing pathways of cellular metabolic flexibility through literature review and querying the WikiPathways database [16]. We started with biochemical reactions involved in glucose and fatty acid oxidation in the adipose tissue, namely the glycolysis and fatty acid β-oxidation processes, and expanded them to link them to each other via the TCA cycle. Next, rate-limiting enzymes as well as transport, signalling and regulatory proteins were included to expand upon the biochemical processes, along with their respective interactions with other components already in the network. This was followed by the addition of fatty acid synthesis downstream of the TCA cycle as a feedback mechanism to fatty acid β-oxidation. Furthermore, cellular signalling cascades known to affect cellular oxidation were also added.

**Fig. 1:**
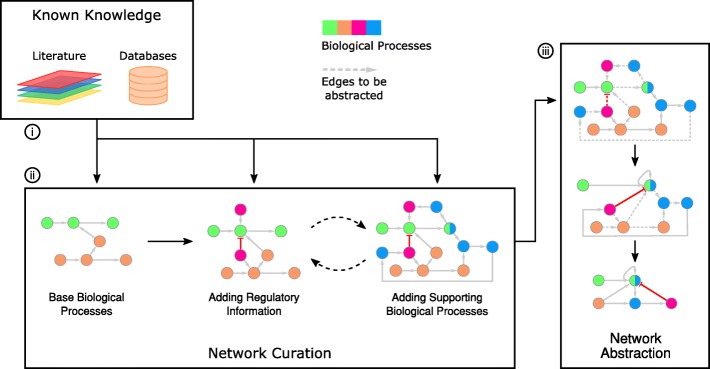
Methodology overview showing the workflow to construct the abstracted network. (i) Known knowledge in the form of published literature and databases is queried regarding cellular metabolism. (ii) Base biological processes are isolated and then expanded by adding regulators and other related processes as long as they are related to cellular metabolism. (iii) The expanded network is then abstracted by merging edges such that only major components and rate-limiting steps remain

Finally, to give a simplified overview and ease its understanding, the network was abstracted by only leaving in rate-limiting steps, major metabolites between the said steps, and associated regulatory proteins. The exact procedure and order of reduction differs from network to network; however, the basic idea remains the same, i.e. to represent multiple nodes and/or edges by a single node and/or edge. Figure 1 illustrates this procedure. As an example, consider the procedure of fatty acid breakdown to release multiple Acyl-CoA molecules, which is a multi-step process involving multiple sets of enzymes and reactions. However, unless we are specifically targeting a step within this procedure, or one of the steps is a rate-limiting step under scrutiny, we can represent the whole breakdown process in an abstracted manner using a fatty acid node, linked to an Acyl-CoA node with an edge.

**Fig. 2:**
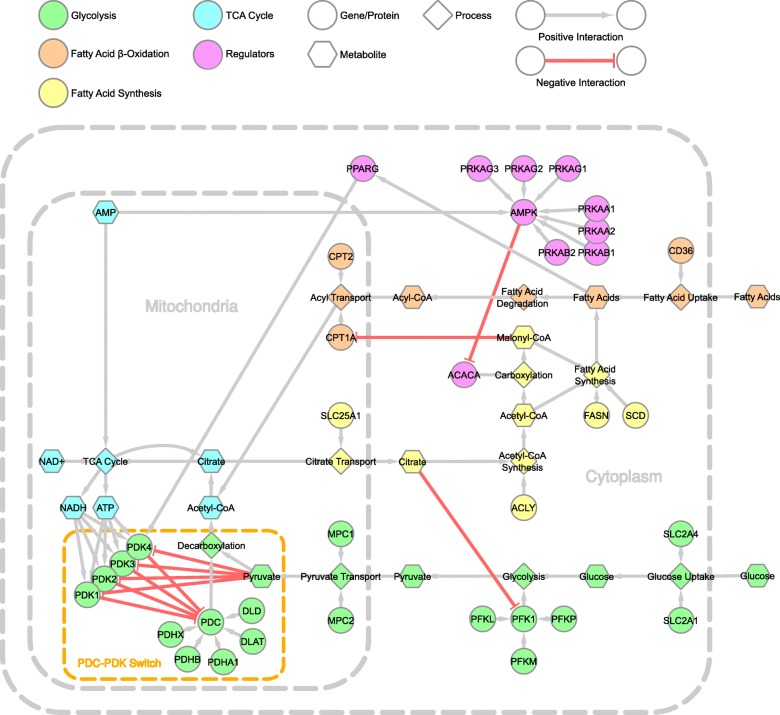
Abstracted cellular network of metabolic flexibility in the adipose tissue. The network consists of different pathways and processes, which are grouped together into five major, colour-coded categories: (i) green for glycolysis, (ii) orange for fatty acid β-oxidation, (iii) yellow for fatty acid synthesis, (iv) cyan for TCA cycle and (v) magenta for regulators of metabolic flexibility

The resultant abstracted cellular network of metabolic flexibility is shown in Fig. 2. The colour coded sections identify the major pathways with, (i) green for glycolysis related components, (ii) orange for fatty acid β-oxidation, (iii) yellow for fatty acid synthesis, (iv) cyan for the TCA cycle and (v) magenta for regulators of metabolic flexibility. In the abstracted network, we also see how these pathways are interacting with each other, in particular how the various products of the TCA cycle are playing roles in activating or inhibiting different pathways through feedback mechanisms. We define any interaction that activates or continues a process in the network as a positive interaction. In the network shown in Fig. 2, these positive interactions cover transcriptional activation, allosteric activation, biochemical reactions (substrate consumption and/or product formation), protein complex formation and species transportation. Negative interactions exclusively refer to inhibitory interactions, whether they are allosteric inhibition or transcriptional inhibition. The following subsections explain these pathways in detail.

### Cellular energy production

The TCA cycle, also referred to as the citric acid cycle or Krebs cycle, is the primary biochemical pathway for cellular energy production and respiration in all aerobic cells [17]. The cycle starts with the conversion of acetyl-CoA into citrate, continues through a series of biochemical reactions where it reduces NAD^+^ to NADH, produces FADH_2_ and CO_2_ and ends with the reconversion to citrate, thus starting the next iteration of the cycle. The NADH and FADH_2_ are then converted into ATPs via oxidative phosphorylation. Thus, by consuming acetyl-CoA, the cycle produces cellular energy in the form of ATPs and replenishes NADH concentrations in the cell. The TCA cycle can be viewed in detail at WikiPathways [16] (pathway ID WP78 [18]) and is shown as the cyan module in Fig. 2. Glucose or fatty acids are consumed upstream of the TCA cycle for the generation of acetyl-CoA, but are regulated by the TCA cycle downstream as well, forming the basis of metabolic flexibility.

#### Glucose uptake and oxidation

Glucose is the most readily utilisable resource for the production of acetyl-CoA for the TCA cycle. The process starts with the uptake of glucose into the cell, which can be insulin-dependent via the *SLC2A4* (also known as *GLUT4*) glucose transporters or insulin-independent via the *SLC2A1* (also known as *GLUT1*) transporters. Glucose is then phosphorylated by phosphofructokinases (*PFK1* and *PFK2*) and converted into pyruvate, a precursor to acetyl-CoA, via glycolysis in the cytoplasm. *PFK1* itself is composed of four subunits consisting of three subtypes: *PFKL* (liver type), *PFKM* (muscle type) and *PFKP* (platelet type), the combination depending on the tissue [1]. The pyruvate converted by the *PFK*s is then transported into the mitochondria by the mitochondrial pyruvate carriers (*MPC1* and *MPC2*), where it is converted to acetyl-CoA via the pyruvate dehydrogenase complex (*PDC*) [19]. The complete pathway including intermediate metabolites and enzymes is available at WikiPathways (pathway ID WP534 [18, 20]), with the abstracted pathway shown as the green module in Fig. 2. Interestingly, *PFK*s are allosterically inhibited by citrate, a primary component of the TCA cycle, when the citrate is transported into the cytoplasm via the citrate carrier (*SLC25A1*) [21, 22].

#### Fatty acid β-oxidation

After glucose, fatty acids, usually in the form of triglycerides, are the preferred source to generate energy via cellular oxidation. The triglycerides are first processed by lipoprotein lipase (*LPL*) outside the adipocytes to yield glycerol and separated fatty acid chains after which the fatty acids are taken up into the cell by fatty acid transporters such as *CD36* [23, 24]. The fatty acid chains are converted into Acyl-CoA by Acyl-CoA synthetase family of enzymes, which is then processed further by various enzymes yielding multiple acetyl-CoA molecules per fatty acid chain (increasing the yield of the TCA cycle per gram of fatty acid) [23, 24]. The rate-limiting step in β-oxidation is controlled by hydroxylacyl-CoA dehydrogenase (*HADH*) for small- and medium-length fatty acids. However, in the adipose tissue, the rate-limiting step is the transport of Acyl-CoA into the mitochondria after the breakdown of long-chain fatty acids, conducted by carnitine palmitoyltransferases (*CPT1A* and *CPT2*) [25]. It has been shown that citrate from TCA cycle can escape into the cytoplasm from the mitochondria, where it is converted to acetyl-CoA by ATP-citrate lyase (*ACLY*), which can then be converted to malonyl-CoA by acetyl-CoA carboxylase (*ACACA*). Malonyl-CoA is known to restrict the uptake of fatty acids into the mitochondria by inhibiting *CPT1A*, thereby redirecting fatty acids towards esterification and storage, and creating a feedback mechanism from the TCA cycle [1]. The complete pathway of fatty acid β-oxidation can be viewed in WikiPathways (pathway ID WP143 [1, 26]), and the abstracted representation is shown as the orange module in Fig. 2.

### Energy storage in the adipose tissue

Excess energy is stored in the form of fatty acids by the conversion of acetyl-CoA into fatty acids by fatty acid synthase (*FASN*) and stearoyl-CoA desaturase (*SCD*) [27]. This conversion can be in response to both higher presence of glucose and the resultant higher output of the glycolytic pathway, and thus, the previously mentioned malonyl-CoA mediated esterification. *FASN* utilises both acetyl-CoA and malonyl-CoA for the production of fatty acids in the cytoplasm, which can then be desaturated by SCD and stored as triglycerides. The cytoplasmic acetyl-CoA can also be provided by the aforementioned cytoplasmic citrate via its conversion by *ACLY*. These fatty acids are then either stored as fat droplets in the adipocytes or are converted into free fatty acids and excreted to be transported to other tissues and organs via plasma albumin [28]. The detailed pathway of fatty acid biosynthesis is available at WikiPathways (pathway ID WP357 [29]), whereas the yellow module in Fig. 2 shows the abstracted representation. Recent studies have cited adverse effects of high quantities of dietary fructose as it has been shown that it promotes de novo lipogenesis, contributing to higher circulating triglycerides, and thus obesity associated chronic diseases [5, 30]. Whether this contribution has any effects on cellular, metabolic flexibility remains to be elucidated.

### The PDC-PDK regulatory switch

In glucose oxidation, *PDC* controls the final step of the conversion of pyruvate to acetyl-CoA for the TCA cycle, and it has been shown that inhibition of *PDC* moves the source of energy production from glucose to fatty acids [31]. *PDC* is composed of three subunits: E1, E2 and E3. Subunit E1 is composed of pyruvate dehydrogenase E1 component subunit alpha (*PDHA1*) and pyruvate dehydrogenase E1 component subunit beta (*PDHB*). Subunit E2 consists of dihydrolipoyllysine-residue acetyltransferase (*DLAT*) while dihyrolipoyl dehydrogenase (*DLD*) comprises subunit E3. Finally, pyruvate dehydrogenase protein X component (*PDHX*) anchors the E2 and E3 subunits together, forming functional *PDC*. One of the major regulators of *PDC* is the pyruvate dehydrogenase kinase (*PDK*) family of proteins which have been shown to deactivate the functioning of *PDC* by phosphorylating it at specific positions [31]. To date, four *PDK* isoenzymes (1–4) have been identified [31, 32]. Of these, *PDK2* and *PDK4* have been found to be ubiquitously expressed, especially in tissues and organs with high glucose and fatty acid oxidation rates, for example the adipose tissue, liver, heart and other muscle tissues [27]. The adipose tissue has been shown to have a dominant expression of *PDK4*. The myocardium, on the other hand, expresses *PDK1* leading to a stricter regulation of *PDC* [1]. *PDK3* expression is the most restricted and has been found predominantly in the brain, testes and kidneys only [1, 31].

In the adipose tissue, the expression of *PDK4* has been shown to regulate the conversion of pyruvate into acetyl-CoA by inhibiting *PDC* activity [27]. However, *PDK4* is allosterically inhibited by pyruvate when in high concentrations, creating a feedback mechanism [27]. In conditions where glucose concentrations drop, less malonyl-CoA is available from the TCA cycle to mediate esterification of fatty acids, allowing fatty acids to be converted into acetyl-CoA via β-oxidation [1]. These effects place the *PDC-PDK4* protein interaction as a substrate switch, effectively changing the energy source from glucose oxidation to fatty acid β-oxidation. Thus, the switch regulates which energy source to metabolise with regard to the nutrient state (glucose or fatty acid availability), as well as whether to focus efforts towards energy production or storage. In addition to pyruvate allosteric inhibition, *PDK4* has been shown to have other allosteric interactions with ATP and NADH inducing *PDK4*-mediated inhibition of *PDC*. These interactions are highlighted visually by the orange dashed box in Fig. 2.

### PPARγ signalling and regulatory effects

In addition to the allosteric interactions, *PDK*s are also regulated transcriptionally via the transcription factors forkhead box protein O (*FoxO*), peroxisome proliferator-activated receptors (*PPAR*s) and oestrogen-related receptor α (*ERRα*) [27]. Of particular interest in the context of the adipose tissue is *PPARγ* and PPARγ coactivator 1α (*PGC1α*) expressions as they affect the transcription of *PDK4*, improving its expression [33, 34]. *PPARγ* is also a cellular fatty acid sensor having a subset of free fatty acids as its ligands [27, 35] and is primarily associated with adipogenesis [33]. *PPARγ* is shown as part of the magenta module in Fig. 2.

### AMPK-mediated override

Under unstressed conditions, either glucose or fatty acids are exclusively utilised as substrate for the TCA cycle. Recently, however, it has been shown that under conditions pertaining to energy stress, caused by either nutrient deprivation or exercise and physical activity, this substrate exclusivity is overridden by the AMP-activated protein kinase (*AMPK*) signalling cascade, allowing both glucose and fatty acids to be utilised for energy production [1]. This override is possible as *AMPK* relies on high concentration of accumulated AMP relative to ATP for its activity. *AMPK* is a heterotrimeric sensor for cellular energy homeostasis, consisting of a catalytic component (α1 or α2), and two regulatory components (β1 or β2; γ1, γ2 or γ3) [36]. The subcomponents α1, α2, β1, β2, γ1, γ2 and γ3 are respectively referred to as *PRKAA1*, *PRKAA2*, *PRKAB1*, *PRKAB2*, *PRKAG1*, *PRKAG2* and *PRKAG3* in Fig. 2. It has been shown that under nutrient stressed conditions, *AMPK*-mediated inactivation of *ACACA* and indirect activation of *PFK* remove the inhibition of glucose oxidation and allow pyruvate to contribute to acetyl-CoA formation [1]. *AMPK*, its subunits, and *ACACA* are collectively shown as part of the magenta module in Fig. 2.

## Mapping gene expressions in metabolic flexibility

Our abstracted cellular network of metabolic flexibility (Fig. 2) can be used as a network resource to visualise and analyse expression data for a quick overview of the expression levels of key factors in metabolic flexibility. To demonstrate this utility, we process and visualise a baseline and a publicly available obesity study dataset separately. The cellular network of metabolic flexibility with the visualised data is provided as Additional file 1 for use as a network resource. Additionally, we opted to retain the genes/proteins usually known to have negligible expression in the adipose tissue in the network to see if their expression is affected in the obesity dataset.

**Fig. 3:**
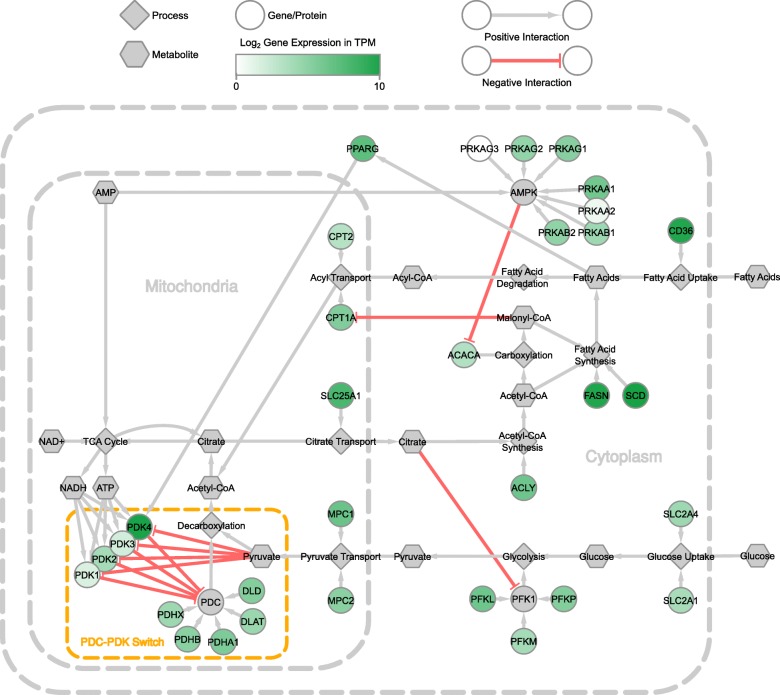
Baseline gene expression of the network in the adipose tissue. Expression is median log_2_ TPM expression of GTEx *Homo sapiens* baseline dataset from Expression Atlas

The log_2_ baseline expression in transcripts per million (TPM) [37] for the adipose tissue is visualised in Fig. 3 using the Genotype Tissue Expression (GTEx) *Homo sapiens* baseline datasets from Expression Atlas (array express ID E-MTAB-5214; [14]). The GTEx project analyses global RNA expression from RNA-seq data of non-diseased tissue from humans and provides a reference of baseline measurements of human gene expression and regulation. The expression trends in the baseline expression correspond to the expression patterns reported in the studies cited earlier, for example the predominant expression of *PDK4* and *PRKAA1* reported in adipose tissue [1, 27].

**Fig. 4:**
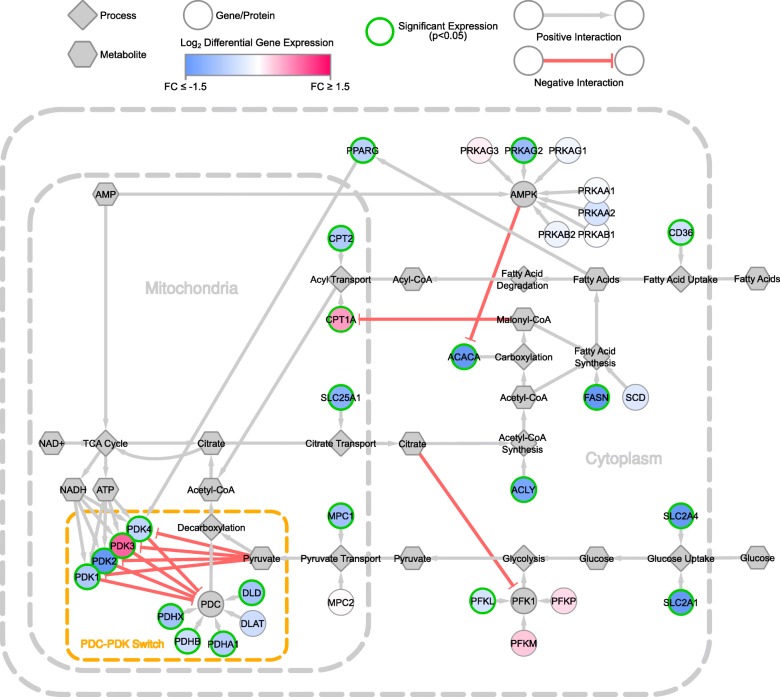
Differential expression of the metabolic flexibility network between obese vs lean healthy individuals. Data shown is GSE55200 from the gene expression omnibus. FC means fold change

Figure 4 shows the log_2_ fold changes in the cellular network of metabolic flexibility in obese individuals as compared to lean individuals. For this visualisation, we used a relatively recent publicly available dataset (GSE55200 [15]) which contains the subcutaneous adipose tissue transcriptomics (microarray) expression from 7 lean and 16 obese individuals. The original study collected the tissue samples from lean healthy, metabolically healthy obese and metabolically unhealthy obese individuals to examine the differences in expression between the groups. In the visualisation, the metabolic processes appear to be impaired in the obese individuals compared to lean individuals, primarily because most genes are being downregulated. The *PDC-PDK* switch is also affected in the obese individuals compared to lean; although, *PDK3* expression is increased in the obese individuals (while those of other *PDK*s are decreased). This seems peculiar considering *PDK3* has little expression as per the GTEx baseline expression dataset, indicating that some signalling or other processes are targeting *PDK3* in obese individuals. These signalling processes targeting *PDK3* can then be further explored or extended in the network for mathematical modelling (using quantitative or qualitative data from the visualised dataset), testing various hypotheses regarding the expression of *PDK3* in obesity, which can then be validated using wet-lab methods. In addition, the glucose and fatty acid transporters also appear to be affected, again signalling a possible impairment of the metabolic processes involved with energy production and homeostasis.

## Conclusions

Our abstracted cellular network of metabolic flexibility highlights the key components involved in metabolic flexibility, providing a resource for directed pathway and system dynamic analyses in the future. Considering the complex interplay between the various cellular processes associated with metabolic flexibility, it is clear that metabolic flexibility is affected in obesity and associated comorbidities, in particular the *PDC-PDK* switch governing the substrate utilisation in cellular respiration. Thus, the cellular network of metabolic flexibility allows us to target various components (enzymes, biological processes, etc.) for further analyses in the context of obesity and the development of chronic diseases.

## Additional file

Additional file 1: The cellular network of metabolic flexibility with the visualised data (7Z 42 kb)
